# Confronting polypharmacy and social isolation in elderly care: a general practitioner’s perspective on holistic primary care

**DOI:** 10.3389/fragi.2024.1384835

**Published:** 2024-05-31

**Authors:** Waseem Jerjes, Daniel Harding

**Affiliations:** ^1^ Research and Development Unit, Hammersmith and Fulham Primary Care Network, London, United Kingdom; ^2^ Faculty or Medicine, Imperial College London, London, United Kingdom

**Keywords:** polypharmacy, social isolation, frail adults, elderly medicine, frailty

## Introduction

In our roles as general practitioners, we frequently encounter the complex issues of polypharmacy and social isolation, especially among our elderly patients (Svensson et al., 2023). The interconnected nature of these challenges underscores the critical need for a holistic approach in primary care (Siqeca et al., 2022). As our society ages, we are seeing an increase in patients with multiple chronic conditions, which often leads to polypharmacy. Concurrently, social isolation is emerging as a significant concern with profound health consequences (Masnoon et al., 2017). The potential interplay between these two factors is gaining recognition in the medical community, prompting a deeper exploration of their combined impact on health (Davies et al., 2020). By adopting a comprehensive care strategy, we have the opportunity to significantly enhance the health and wellbeing of our patients, addressing not just their medical needs but also the social determinants of health that significantly influence their overall quality of life.

## Polypharmacy

Polypharmacy, defined as the concurrent use of five or more medications, is becoming a staple in the treatment regimens of our aging patients (Delara et al., 2022). Both empirical research and clinical observations highlight the dangers associated with polypharmacy, including adverse drug reactions, falls, potential drug interactions, and a heightened likelihood of hospital admissions (Masnoon et al., 2017; Svensson et al., 2023). In general, polypharmacy could be seen as exacerbating the physical effects of aging. In addressing the issue of polypharmacy, particularly in the context of chronic diseases and multimorbidity, general practitioners navigate increasingly complex health landscapes (Jarling et al., 2022; Siqeca et al., 2022).

## Social isolation

The phenomenon of social isolation among the elderly is characterized by a tangible absence of social connections, as opposed to the subjective sensation of loneliness, and is linked with increased risks of morbidity and mortality (D’cruz and Banerjee, 2020). This condition emerges from a complex interplay of physical, psychological, and social factors, which can all negatively alter health behaviours (Leigh-Hunt et al., 2017). The ramifications of social isolation can deeply impact the psychological realm, often precipitating conditions such as depression, anxiety, and cognitive decline, exacerbating the psychological challenges associated with aging (Barton et al., 2014).

## The interplay between polypharmacy and social isolation

There is increasing interest in the interplay and compounding effects of polypharmacy and social isolation. Moreover, the isolation experienced by many elderly individuals exacerbates the challenges posed by polypharmacy (Table 1). Social isolation not only effects mental and emotional wellbeing but also has tangible impacts on how individuals manage their health. For example, a lack of social support can lead to irregular medication intake, missed appointments, worsened health behaviours, and a general decline in health vigilance. Patients who are socially isolated have been found to exhibit suboptimal medication compliance and overall health management, which further exacerbates their physical health conditions (Siqeca et al., 2022; Svensson et al., 2023). This scenario underscores the importance of incorporating social care into the management plan for elderly patients (de Jong Gierveld and van Tilburg, 1999; Leigh-Hunt et al., 2017; Masnoon et al., 2017).

**TABLE 1:** Challenges and strategies in managing polypharmacy among the elderly.

Challenges in polypharmacy	Management strategies
Increased risk of drug-drug interactions due to multiple medications	Regular medication reviews to evaluate and adjust drug combinations
Side effects leading to decreased quality of life and additional health issues	Implementing deprescribing protocols for unnecessary or harmful medications
Difficulty in adhering to complex medication regimens	Patient education on medication schedules and reminders
Prescribing cascade (adding more medications to treat side effects of others)	Vigilant monitoring for side effects and proactive medication adjustment
Altered pharmacokinetics in elderly affecting drug efficacy and safety	Personalized medication plans considering patient’s age and health status
Risk of drug-disease interactions complicating existing health conditions	Collaborative care with specialists to ensure comprehensive treatment

Addressing the complex web of social isolation and its ramifications on mental health reveals a stark reality. The psychological toll of isolation often manifests as depression, anxiety, and cognitive decline among the elderly, complicating their ability to manage physical health issues (Barton et al., 2014). This interplay between mental and physical health can obscure the root causes of symptoms, as signs of mental health struggles might be mistaken for adverse reactions to medications, and *vice versa*. Such diagnostic challenges underscore the need for a holistic approach that encompasses both medical and social considerations in patient care. This diagnostic landscape also underscores the necessity for a holistic healthcare approach that integrates medical and social considerations to effectively address the intertwined challenges of polypharmacy and social isolation (Leigh-Hunt et al., 2017; Siqeca et al., 2022).

## Proposed interventions

The coexistence of polypharmacy and social isolation presents a distinctive set of challenges, with interventions that fail to simultaneously tackle both being much less likely to succeed in our experience, whilst also having more challenge for the clinician in getting “buy in” from the patient. This necessitates a healthcare approach that extends beyond conventional medical treatments to encompass a broader spectrum of patient care (Davies et al., 2020).

In addressing polypharmacy within the elderly demographic, it becomes clear that the traditional focus on medication management alone is insufficient. The complexities of chronic conditions in these patients often result in a labyrinth of prescriptions, each aiming to tackle a specific aspect of their health (D’cruz and Banerjee, 2020; Cooper et al., 2015). However, this fragmented approach overlooks the critical interdependencies within an individual’s health profile. For instance, the clear understanding of how medications interact not just with each other but also with the patient’s lifestyle, diet, and other non-pharmacological factors is often underappreciated (de Jong Gierveld and Havens, 2004; Masnoon et al., 2017). This gap in care necessitates a shift towards a more integrative medication management strategy, one that harmonizes pharmaceutical and non-pharmaceutical interventions. By doing so, we can enhance the efficacy of medical treatments while minimizing the adverse effects that stem from polypharmacy.

Furthermore, it is imperative that primary care physicians adopt a proactive and meticulous approach towards medication management. This involves conducting thorough medication reviews that go beyond mere prescription oversight to include a deep understanding of each patient’s unique health profile, lifestyle, and social environment (D’cruz and Banerjee, 2020; Gnjidic et al., 2012). Such reviews should aim to optimize therapeutic outcomes by minimizing unnecessary medications and reducing the risk of adverse effects, thereby enhancing patient safety and wellbeing. The goal is to ensure that each medication serves a definitive purpose, aligns with the latest clinical guidelines, and contributes positively to the patient’s overall health strategy.

Given how social isolation interacts with polypharmacy, it is also crucial to integrate social care into the medical management plan (Table 2). This requires a concerted effort to identify and understand the social determinants of health that affect elderly patients, such as living conditions, access to community resources, and the strength of their social networks (Siqeca et al., 2022). By fostering strong collaborations with social care providers and community organizations, primary care physicians can facilitate access to supportive services and social activities that promote engagement and connectivity (Davies et al., 2020). These can include community health programs, support groups, and technology-based interventions designed to reduce loneliness. This holistic approach not only addresses the immediate health concerns, such as medication adherence and overall health management, but also contributes to the broader objective of improving the quality of life for elderly individuals, making healthcare more responsive, compassionate, sustainable, and tailored to the unique needs of this vulnerable population (Cooper et al., 2015; Leigh-Hunt et al., 2017; Jarling et al., 2022).

**TABLE 2:** Impact of social isolation on elderly health and intervention approaches.

Health impact of social isolation	Intervention approaches
Mental health issues like depression and anxiety	Access to mental health services, including counselling and therapy
Increased risk of chronic physical conditions	Community health initiatives promoting physical activity and health monitoring
Decreased adherence to medical treatments and medications	Telemedicine to provide regular follow-ups and medication management
Cognitive decline and reduced mental acuity	Social engagement activities like group classes or memory exercises
Heightened sense of loneliness and reduced life satisfaction	Programs to encourage social interactions, such as community events or clubs
Physical health deterioration due to lack of support	Integrated care models involving family, community, and healthcare providers

Expanding on this, it becomes evident that the management of polypharmacy must be intertwined with an understanding of the patient’s social backdrop. Embracing a multifaceted strategy in primary care, particularly for the elderly, requires general practitioners to assess not just the clinical aspects but also the socio-environmental factors impacting patient health, including social networks and wellbeing (Fried et al., 2014; Garvey et al., 2015; Wood et al., 2021). Regular, thorough evaluations that encompass physical health assessments, medication management, and psychological support are fundamental. The interrelation between an individual’s social environment and their health status is undeniable, necessitating a broader approach that includes or expands regular social needs assessments within existing frailty or comprehensive geriatric assessments (Robles Bayón and Gude Sampedro, 2014; Delara et al., 2022). By identifying and proactively addressing these social determinants of health, we can better tailor our interventions to meet the holistic needs of our patients, enhancing their overall health and wellbeing. These can feed into collaborative care with pharmacists, mental health professionals, social workers, and community groups, as part of developing a comprehensive care plan. Moreover, leveraging social prescribing and community resources can proactively and significantly enhance the holistic wellbeing of patients.

In the practice of primary care, our role extends beyond the mere management of medications; it involves actively engaging with our patients to foster an environment of health literacy and autonomy (Svensson et al., 2023). This should extend to patient discussions about the problems of polypharmacy, elucidating its potential risks and impacts on daily living, while simultaneously highlighting the value of maintaining robust social connections (Masnoon et al., 2017). Through educational initiatives and personalized conversations, we strive to elevate our patients’ understanding of their health conditions and treatment plans (Leigh-Hunt et al., 2017). This empowers them to make informed decisions about their health, leading to improved adherence to prescribed therapies and a proactive stance towards nurturing their social wellbeing. Patients themselves are also best placed to make decisions about enhancing their social networks, within their personal, family, and cultural context.

Moreover, the integration of innovative care strategies, such as shared decision-making and patient-centred care planning, marks a pivotal shift towards a more inclusive and participative model of healthcare (Vyas et al., 2021). By involving patients and their caregivers in the decision-making process, especially in the context of managing multiple medications and navigating the complexities of their social environments, we not only respect their autonomy but also enhance their sense of control over their health outcomes (Leigh-Hunt et al., 2017; Delara et al., 2022). This collaborative approach, underpinned by mutual respect and open communication, fosters a therapeutic partnership between patients and healthcare providers (Wimmer et al., 2017). For example, where open dialogue about patient experiences of medication harm or non-adherance is encouraged, healthcare providers can better understand their patients’ perspectives and tailor their approaches to meet individual needs (Garvey et al., 2015). It is within this partnership that we can truly address the multifaceted challenges of polypharmacy and social isolation, ultimately leading to a more holistic and satisfying healthcare experience for our elderly patients (Delara et al., 2022).

Addressing the prescribing cascade, a phenomenon where new medications are introduced to manage side effects from existing treatments, is a critical aspect of managing polypharmacy (Chen et al., 2023). This cycle often exacerbates the medication burden on patients, particularly the elderly, leading to increased risks of adverse drug reactions and further complicating their care. Proactive measures such as de-prescribing, where unnecessary medications are systematically discontinued, can play a pivotal role in alleviating this burden. Implementing such strategies requires a careful balance, ensuring that patients continue to receive essential treatment while minimizing the risks associated with excessive medication use.

Additional focus is needed on fostering strong partnerships with community resources, to amplify our efforts in combating the adverse effects of polypharmacy and social isolation (Garvey et al., 2015). Establishing robust networks with local community centres, support groups, and charities can provide our patients with more accessible, comprehensive care. These alliances play a critical role in enhancing social connectedness and enhance holistic care (D’cruz and Banerjee, 2020; Leigh-Hunt et al., 2017; Wood et al., 2021). By integrating these resources into our care plans, we can offer more personalized, effective solutions that address the wide array of challenges our elderly patients face, ultimately leading to improved health outcomes and a higher quality of life. Use of either local community interventions, or alternatively online interventions, further ensures these benefits can reach those with mobility or access needs.

Addressing polypharmacy and social isolation within healthcare systems demands innovative technological and policy-driven solutions, mindful of existing constraints. Strategic use of digital health tools can enhance patient monitoring and medication management, while policies fostering community engagement and interdisciplinary collaboration can mitigate social isolation impacts (Wimmer et al., 2017; Wood et al., 2021). Tailoring these interventions to fit within the operational and budgetary realities of healthcare systems is essential for sustainable implementation and maximized patient benefit. Furthermore, advocating for policies that support holistic care models at both national and international levels can bring about systemic changes beneficial for the elderly.

## Conclusion

In conclusion, the dual challenges of polypharmacy and social isolation in primary care necessitate a holistic, multidisciplinary approach. Integrating medical, psychological, and social care, while also considering broader systemic and policy-based interventions, can significantly improve patient outcomes and enhance the quality of life for our elderly population, whilst limiting the detriments associated with advancing age. As general practitioners, we are positioned uniquely to lead this change, drawing upon our diverse experiences and perspectives to deliver comprehensive and empathetic care.
